# Evaluation by the Ames Assay of the Mutagenicity of UV Filters Using Benzophenone and Benzophenone-1

**DOI:** 10.3390/ijerph15091907

**Published:** 2018-09-02

**Authors:** Wen-Qian Wang, Hai-Xin Duan, Zhou-Tao Pei, Rou-Rou Xu, Ze-Tian Qin, Guang-Can Zhu, Li-Wei Sun

**Affiliations:** 1 School of Energy & Environment, Southeast University, Nanjing 210096, China; 220160595@seu.edu.cn (W.-Q.W.); 15895918358@163.com (H.-X.D.); 220170623@seu.edu.cn (Z.-T.P.); zt746603025@gmail.com (R.-R.X.); skyxiaoyaoyou@gmail.com (Z.-T.Q.); gc-zhu@seu.edu.cn (G.-C.Z.); 2 Taihu Lake Water Environment Engineering Research Center (Wuxi), Southeast University, Wuxi 214000, China

**Keywords:** UV filters, benzophenone, benzophenone-1, mutagenicity, genotoxicity, Ames assay

## Abstract

Ultraviolet absorbing chemicals (UV filters) are widely used in personal care products for protecting human skin and hair from damage by UV radiation. Although these substances are released into the environment during production and consumption processes, little is known about their genotoxicity effects. Our previous studies have shown that benzophenone-type UV filters exhibited acute toxicity on three species of aquatic organisms. Mutagenesis by benzophenone (BP) and benzophenone-1(BP-1) was tested in the present study by the *Salmonella typhimurium*/reverse mutation assay (Ames assay). All the positive reverse mutations occurred in the absence of the S9 liver extract system for both chemicals. From BP, positive mutation effects on the TA102 strain at doses of 0.05 μg/plate and 0.5 μg/plate were detected. From BP-1, positive mutation effects on the TA97 strain at doses of 0.05 μg/plate and 0.5 μg/plate, and on the TA100 strain at a dose of 0.5 μg/plate, were detected. A mixture of BP and BP-1 exhibited mutagenicity on the TA97 and TA100 strains. For the TA97 strain, the positive mutation results were detected at 10% and 50% of the mixture. For the TA100 strain, the results were detected when the mixture was at 5% and 10%. In the mixture at 5%, the concentrations of BP and BP-1 were 3.5 μg/plate and 14 μg/plate, respectively. In the 10% mixture, the doses of BP and BP-1 were 7 μg/plate and 28 μg/plate, respectively. In the 50% mixture, the doses of BP and BP-1 were 35 μg/plate and 140 μg/plate, respectively. The mixture test results suggested that there was antagonism in mutagenicity between BP and BP-1.

## 1. Introduction

With the wide use of personal care products (PCPs), exposure to chemicals from PCPs in the environment has gradually attracted the attention of researchers. Within the PCPs, ultraviolet absorbing chemicals (UV filters) is a new product group in the cosmetics field. UV filters protect human skin and hair from radiation harm. UV radiation is a risk factor for cataract and macular degeneration [1]. Almost all sunscreens contain benzophenone compounds. As a major class of UV filters, benzophenone-type UV filters have been widely used due to the excellent absorption properties and low price [2]. Therefore, the use of benzophenone in the cosmetics field has continued to expand to conditioners, lotions, and lipsticks. In addition, it is a spice fixative that imparts a sweet aroma and is used in many perfumes and soaps [3]. In the production and daily consumption processes, the residues of UV filters are thought to be largely discharged into wastewaters by the production process and daily use [4].

Despite the high efficiency of the current sewage treatment process, the existing domestic sewage treatment facilities cannot effectively treat UV filters, and there is still great uncertainty in the removal effect [5]. Furthermore, until now, there are still no regulations or emission standards for manage UV filters [5]. As a result, PCPs and their metabolites have been frequently detected in the environment.

A study conducted in Spain measured the concentration of UV filters in 51 water samples. The maximum concentration of benzophenone (BP)-1 was 1.2 ng/L, BP-2 was 27.2 ng/L, BP-3 was 15.2 ng/L, and BP-8 was 21.6 ng/L [6]. For the first time in 2013, the presence of BP-1 was detected in indoor dust [7]. The survey respondents included China, the United States, Japan, and South Korea, where the concentrations were 7.38, 102, 10, and 26.2 ng/g, respectively.

In the past, benzophenones (BPs) have been considered harmless. However, toxicological studies [3,4,8,9,10,11] have shown that BPs have certain acute toxicities, genotoxicities, reproductive and endocrine disruptive effects, as well as significant estrogenic activity.

Despite these studies, there are still no genetic toxicity studies about BPs. Genotoxicity tests detect genetically damaging substances by different mechanisms in vitro and in vivo by DNA damage, and its fixation can be detected, which is one of the keys to the development of malignant tumors.

Studies about the genotoxicity of BPs are primary screenings for carcinogens and an important means toward preventing cancer in humans. Benzophenone contains about twelve derivatives, designated as benzophenone-1 through benzophenone-12, and all of them are widely used in cosmetic products as a photostabilizer, and as a sunscreen in lotions and hair sprays [12]. With the popularity of sunscreen products, the effects of long-term and low-dose exposure to BP on the human body are worth noting. A growing number of studies have demonstrated that BPs can interfere with the endocrine system and, therefore, have been classified as chemicals suspected of having endocrine disrupting effects [13]. In vivo and in vitro studies have shown that BP-1 exhibits estrogenic and anti-androgenic activities [14]. High concentrations of BP-1 in urine may cause estrogen-dependent diseases, such as endometriosis in women [15].

Although BP in the environment is at a low concentration, long-term emissions and potential hazards in the water environment need to be investigated. Only a small number of related studies have been conducted on BPs [16,17,18].

There are still no detailed reports about the mutagenicity of BPs. Similar research by Kozumbo [18] reported that dimethyl phthalate (DMP) produces chromosomal aberrations in rat liver following sub-chronic application of phthalate to the skin. It produced in the bacterial TA100 tester strain a dose-related mutagenic response that was abolished by NAD- and NADP- independent metabolism associated with rat liver microsomal preparations (S9).

In this study, the mutagenicity of BP and BP-1 was evaluated by *Salmonella typhimurium* /reverse mutation assay (Ames assay) in order to explore the potential genetic hazards. Furthermore, because the Ames assay can detect mutagenicity of multiple mixed pollutants quickly and accurately [19,20,21], the effects of mixed BP and BP-1 were also investigated. In our previous acute toxicity studies [3,4,11], it has been confirmed that the different combinations of different BP substances exhibited different effects, as additive effects or antagonistic effects. The mutagenic effects of their mixtures further explored the mechanisms of BP chemicals. The results will provide a scientific basis for formulating environmental criteria and further protecting of human health.

## 2. Materials and Methods

### 2.1. Chemicals

Analytical grade BP (CAS:119-61-9) was purchased from Macklin Biochemical Co., Ltd., (Shanghai, China). BP-1 (CAS:131-56-6) at 99% purity was purchased from Aladdin Biochemical Technology Co., Ltd., (Shanghai, China). Analytical purity grade dimethyl sulfoxide (DMSO) (Sinopharm Chemical Reagent Co., Ltd., Shanghai, China) was used as a solvent.

NaN_3_, Dexon, Dantron, and 2-AF at 99% purity were purchased from Maclean Biochemical Technology Co., Ltd., (Shanghai, China), which were used as positive mutagenicity controls. Liver microsomal S9 was purchased from CHI Scientific, Inc., (Jiangsu, China).

### 2.2. Salmonella Typhimurium Strains for the Ames Assay

TA97, TA98, TA100, and TA102 were used as standard test strains [19,20,21]. The strains were provided by Jiangsu Provincial Center for Disease Control and Prevention and Guizhou Medical University. TA97 and TA98 strains primarily detect the genotoxic substances of frameshift mutants, while TA100 and TA102 strains detect DNA as a mutagenic substance for base pair substitution [20,21]. TA102 strain has a different specificity from the other Salmonella strains routinely used in mutagenicity screening. It detects a variety of oxidative mutagens and seems to be more sensitive to some aldehydes and some DNA damaging compounds, which are negative with other Salmonella strains [22].

### 2.3. Certification Test of the Characteristics of the TA Strains

The characteristic identification tests were carried out to certify the quality of the strains. The tests included histidine deficiency, lipopolysaccharide barrier defect, ampicillin resistance, UV sensitivity, tetracycline resistance, spontaneous reversion, and reversible characteristics, a diagnostic test. All tests were carried out according to the standard Ames bacterial mutagenicity assay [19].

### 2.4. Bacterial Inhibition Experiment of BP and BP-1

Bacterial inhibition experiment is necessary according to the standard Ames bacterial mutagenicity assay [20,21]. Safety and Technical Standards for Cosmetics require that the maximum chemical dose should be 5 mg/plate [19]. DMSO was used as a solvent to configure BP and BP-1 solutions. The maximum concentration of both were 50 mg/mL, then diluted to 25 mg/mL, 10 mg/mL, 5 mg/mL, and 0.5 mg/mL. Plates were poured with LB medium, and 0.1 mL of the above solution and 0.1 mL of bacterial solution per dish were added.

The criteria for determining the highest dose of the test substance are its toxicity to the bacteria and its solubility. A decrease in spontaneous reversion variables, a decrease in background bacteria, or a decrease in the number of bacteria in the culture being treated are signs of toxicity [19].

### 2.5. Ames Assay

According to the standard Ames bacterial mutagenicity assay [20,21] and Safety and Technical Standards for Cosmetics [19], the experiments were performed by the plate incorporation method with or without liver microsomal metabolic activation (S9) mixture. For the pure materials, the highest dose group was generally 5 mg/plate or 5 μL/plate. After the bacterial inhibition experiment, five dose groups (0.05, 0.5, 5, 50, 500 μg/plate) were determined for testing the chemicals. Three parallel plates were prepared for each dose. The chemicals (test chemicals or positive control drugs), the S9 system (with or without), and the *S. typhimurium* strains (TA97, TA98, TA100, TA102) were added to the nutrient mediums. The negative control (blank and solvent) groups and the positive control groups were also done with three parallel plates.

Positive control experiments were conducted to demonstrate the sensitivity of the strains. The chemicals and concentrations were as follows: In the absence of the S9 system: Dexon 50 μg/plate for the TA97, TA98, and TA102 strains, and NaN_3_ 1.5 μg/plate for the TA100 strain. In the presence of the S9 system: 2-AF 10 μg/plate for TA97, TA98, and TA100 strains, and Dantron 50 μg/plate for the TA102 strain [19,20,21].

Five concentrations for each chemical were tested for mutagenicity in the standard plate pre-incubation assay. The reported numbers of reverse mutation colonies per plate are mean values from triplicates obtained in one experiment. The experiment was repeated at two times.

All the plates for the experiments were incubated under the same conditions: 37 ± 0.5 °C, 48 h, dark.

### 2.6. Mixed BP and BP-1 for the Ames Assay

Referring to the reported concentrations from environmental and the previous acute toxicity test results [3,4,5,11,12], the mixing ratio and concentrations of BP and BP-1 were determined based on the results of separating tests. BP and BP-1 were mixed at a 1:4 concentration. Stock solutions of BP and BP-1 were prepared at 70 and 280 mg/L, respectively. The test series was set as 100%, 50%, 10%, 5%, and 0.5%. The concentration ranges were (BP + BP-1): 100% (70 μg/mL + 280 μg/mL), 50% (35 μg/mL + 140 μg/mL), 10% (7 μg/mL + 28 μg/mL), 5% (3.5 μg/mL + 14 μg/mL), and 0.5% (0.35 μg/mL + 1.4 μg/mL). Then, 100 μL of the mixtures, which were at different doses, was added to each plate.

Testing a mixture of BP and BP-1 was conducted with the same method as for the separate compounds.

### 2.7. Analysis

The mutagenicity ratio (MR) is calculated as

MR = x/x_0_ (1)

where x is the number of reverse mutation colonies on compound-treated plates and $x_{0}$ is the number of reverse mutation colonies in the negative control group.

When the mutagenicity ratio (MR) is ≥2 and the dose–response relationship and the background are normal, the mutagenesis is judged as positive.

The significant difference between the spontaneous mutation colonies in the blank and the reverse mutation colonies in the DMSO control group were carried out by the *t*-test.

## 3. Results

### 3.1. Negative Control and Positive Control Results

Table 1 is the results of reverse mutation colonies in the negative control groups (including blank and DMSO control) and positive control groups. The spontaneous mutation colonies in the blank from the four strains were all in the reasonable numbers. There were no significant differences (*t*-test, *p* < 0.01) between the spontaneous mutation colonies in the blank and the reverse mutation colonies in the DMSO control group, which proved that DMSO did not cause mutations at the doses used in the present study.

All the positive control groups caused significant mutations in the four strains. According to the Ames bacterial mutagenicity assay [19,20,21], the reverse mutation colonies from the four strains were all in reasonable numbers, which indicated that the four strains used in the present study are qualified for the Ames assay. The results proved that the four strains and the experimental system are credible.

### 3.2. Ames Assay of BP

**Figure 1:**
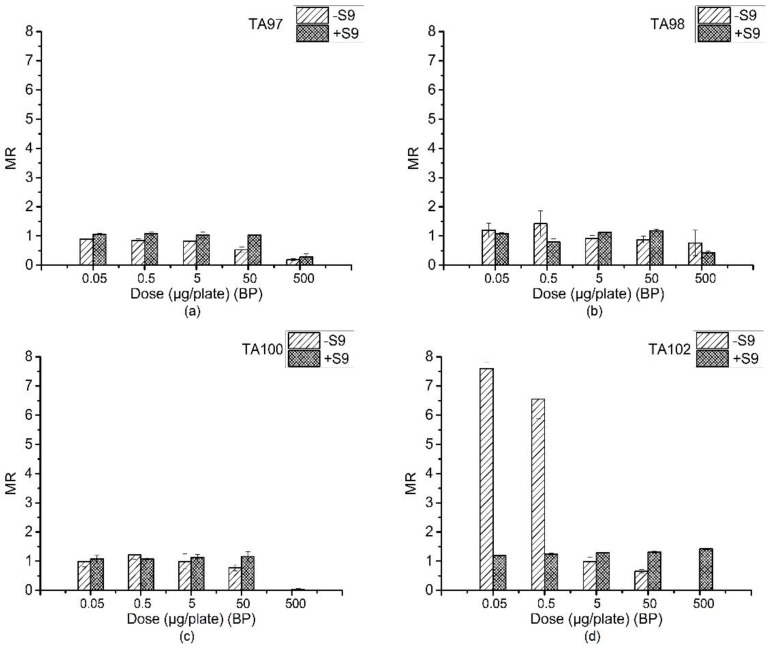
Mutagenesis of four strains by BP in the presence and absence of S9 liver extract; (**a**) TA97 strain; (**b**) TA98 strain; (**c**) TA100 strain; (**d**) TA102 strain. The mutagenicity ratio (MR) is the average ratio (±SE) from three parallel experiments.

Table 2 is the reverse mutation colonies of four *S. typhimurium strains* detected by BP with the Ames assay. The MR values at every dose in both the +S9 and −S9 systems are compared in Figure 1.

For the TA97 strain, as shown in Figure 1a, in the system without S9, the MR decreased as the dose increased and even dropped to 0.28 when the dose was 500 μg/plate. The addition of the S9 system had a slight increasing effect on the MR compared to those without S9. From the dose of 0.05 μg/plate to 500 μg/plate, no significant mutagenic activity was detected in the TA97 strain.

For the TA98 strain, the detected mutagenicity result was negative in systems both with and without S9, as shown in Figure 1b. There was no significant increase in the reverse mutation colonies with an increase in any of the concentrations. In contrast, when the dose was 500 μg/plate in the presence of liver microsomal S9, the number of reverse mutation colony was lower than the blank group, which indicated possible inhibition effects on bacteria by BP.

The mutagenicity result of the TA100 strain was also negative and lacked a dose–response relationship according to Figure 1c. Similar to the TA98 strain, when the dose was 500 μg/plate, BP had a strong inhibition effect on the TA100 strain whether or not S9 was added.

Figure 1d shows the results of BP for its mutagenic activity for the TA102 strain. In the absence of S9, when the dose of BP was 0.05 and 0.5 μg/plate, the MR value was greater than 2, which indicated significant mutagenicity of BP to the TA102 strain. However, with an increasing of the dose, the mutagenicity of BP decreased. When S9 was added to the system, MR decreased to below two in all concentrations. It may be that the metabolic activity of the liver caused the mutation to be eliminated [18].

### 3.3. Ames Assay of BP-1

**Figure 2:**
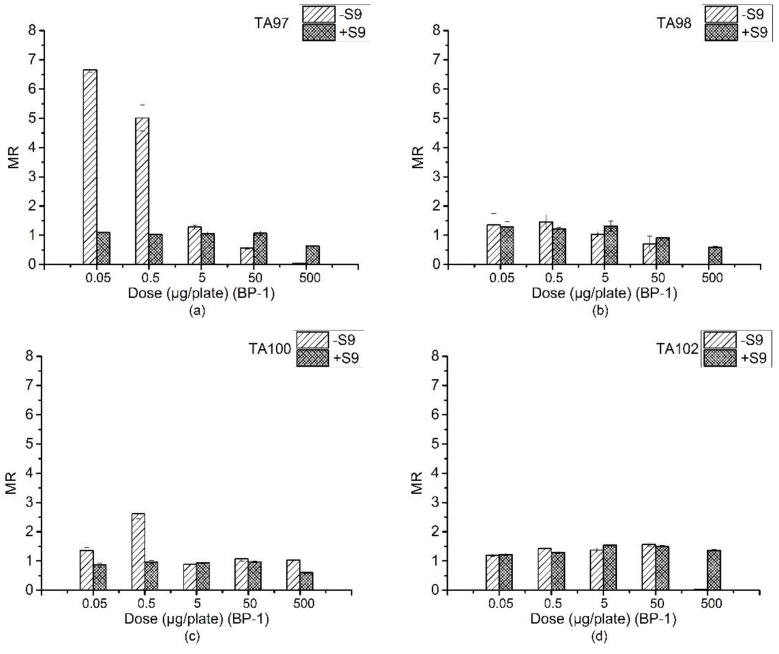
Mutagenesis of four strains by BP-1 in the presence and absence of S9 liver extract; (**a**) TA97 strain; (**b**) TA98 strain; (**c**) TA100 strain; (**d**) TA102 strain. The mutagenicity ratio (MR) is the average ratio (±SE) from three parallel experiments.

Table 3 is the reverse mutation colonies of four *S. typhimurium* strains detected by BP-1 with the Ames assay. The MR values at every dose in both +S9 and −S9 systems are compared in Figure 2.

From Figure 2a, BP-1 had significant mutagenicity on the TA97 strain at doses of 0.05 and 0.5 μg/plate in the absence of S9. However, the mutation rates in the high-dose experimental group decreased. The phenomenon is similar to BP for strains TA100 and TA102. We speculate that it may be due to the strong DNA damage or inhibitory effects for specific *S. typhimurium* strains. In the +S9 system, no mutagenic activities were detected with doses from 0.05 μg/plate to 500 μg/plate, which is similar to the result with BP.

The results of the TA100 strain exposed to BP-1 are shown in Figure 2c. In the absence of S9, MR was greater than 1 but less than 2 at a dose of 0.05 μg/plate. The mutation rate was more than doubled when the dose reached 0.5 μg/plate, exhibited a significant mutation effect. However, there were no obvious mutations at the doses of 5, 50, and 500 μg/plate.

In the presence or absence of S9, BP-1 was not mutagenic to the TA98 and TA102 strains (Figure 2b,d). An increasing number of inverse mutants were observed in the presence and absence of S9 liver extracts from the 0.05 to 50 μg/plate dose of BP-1 in the TA102 strain, but the increase was not significant. The inverse mutants in 500 μg/plate without S9 was lower than those in other doses. It may be an extreme DNA- or cell-level damage that inhibited the growth of the strains.

### 3.4. Ames Assay of Mixtures of BP and BP-1

The Ames assay of a mixture of BP and BP-1 was designed based on the results of the separating Ames mutagenicity assays and the previous acute ecotoxicity tests [3,4,11]. The “low dose” was set according to most environmental human exposure levels [23].

The experimental results are shown in Table 4. Positive results were found in the TA97 and TA100 strains without S9.

For the TA97 strain when the mixture was at 10% and 50%, the MR values were ≥2. For 10%, the concentrations of BP and BP-1 were 7 and 28 μg/plate, respectively. For 50%, the concentrations of BP and BP-1 were 35 and 140 μg/plate, respectively.

For the TA100 strain, MR ≥ 2 was detected when the mixtures were at 5% and 10%. For the 5%, the concentrations of BP and BP-1 were 3.5 and 14 μg/plate, respectively.

For the TA102 strain, reverse mutation colonies were slightly increased compared to the absence of S9 in the case of 5% to 100%. It can be considered to have a weak positive reaction to the TA102 strain.

There was no significant result for the TA98 strain in either the +S9 or −S9 system.

## 4. Discussion

Nakajima et al. studied the mutagenicity of BP and BP-1 for the TA98 and TA100 strains under concentrations of 300 μg/plate and 500 μg/plate [17]. None of the compounds produced clear positive mutagenicity results under any conditions. Their results for TA98 and TA100 strains are similar to ours. There were no reports on the TA97 and TA102 strains. Furthermore, the study indicated that five benzophenones (including BP and BP-1) showed inhibition of bacterial growth at high concentrations. Yamamoto’s [16] study also confirmed the inhibition effect and negative result on the TA98 and TA100 strains of BP.

In our experiment, no mutation was detected from four strains for both BP and BP-1 in the +S9 system. This may conclude that the addition of the S9 activation system led to the elimination of mutations. Yamamoto [16] studied the mutagenicity of BP and its chlorinated products on the TA98 and TA100 strains and proved that the chlorinated product is not mutagenic. A study by Nakagawa [12] determined that BP is enzymatically converted to benzhydrol and p-hydroxybenzophenone, and its sulfate is conjugated in rat hepatocytes. His study supported the results in the present study and explains the no mutation effects in the +S9 system. Although there was no evidence for the enzymatic reaction for BP-1, it may be similar to BP because the chemical structures of the two chemicals are similar. From the previous and the present study, the mutagenicity of the BP series of chemicals is the characteristics of themselves. In contrast, the metabolic activity eliminated the mutation.

The detected mutagenicity types from BP and BP-1 are different: BP caused positive reverse mutations in the TA102 strain. The TA102 strain detected DNA as a mutagenic substance for base pair substitution. BP-1 caused positive reverse mutation in the TA97 and TA100 strains. The TA97 strain only detected the frameshift mutants. The TA100 strain was similar to the TA102 strain, which detected the frameshift mutants for base pair substitution.

The difference in the chemical structure between BP and BP-1 is that BP-1 has two hydroxyl groups on the benzene ring. Other than that, their structures are very similar. In spite of the similar structure, the results of our study indicate a diversity of mutagenicity types of the two chemicals. In the present study, only two kinds of BPs were detected, and mutagenicity was confirmed, which can be predicted that the left BPs and further chemicals used in UV filters may have mutagenicity and the types may be different. Therefore, the mutagenicity of the chemicals needs further studies.

For both BP and BP-1, no mutagenicity was detected when the dose was higher than 5 μg/plate. The inhibition effects of BP and BP-1 in *S.* *typhimurium* strains were supposed. For this, bacterial growth inhibition tests were designed and carried out. The results are shown in Table 5.

From the results (Table 5), when the dose was higher than 500 μg/plate, significant inhibition effects were observed. The higher the dose, the fewer colonies grew on the petri dish. There were even drug precipitates at the highest dose plates. The results may explain why there was no dose–response relationship in this experiment. Usually, MR should increase with an increasing of the tested dose. However, the inhibitory effects on *S.* *typhimurium* strains at high concentrations interfered with the mutagenicity effects. It could be possible that BP and BP-1 caused DNA damage that the bacteria were unable to repair, henceTA strains could not grow andthe mutagenicity effects could not be detected. Although significant inhibition effects were not detected with the concentrations of 5 μg/plate and 50 μg/plate, BP or BP-1 may affect the reverse mutation activity of the strains.

According to the Ames assay of the mixture of BP and BP-1, the results are closely related to the separating tests In the positive mutagenicity results of the mixed experiment, the corresponding doses of BP were 3.5, 7, 35 μg/plate, and the doses of BP-1 were 14, 28, 140 μg/plate. Furthermore, the positive mutations were observed in the TA97 and TA100 strains. Compared with the results of the separating experiments, the dose of the mixture that produced mutations was higher than that in the separate experiments.

For the TA97 strain in the separating test, BP showed negative mutagenicity while BP-1 showed positive mutagenicity at doses of 0.05 and 0.5 μg/plate. In the mixed tests, positive mutagenicity results appeared at two dose levels in which the doses of BP-1 were 28 and 140 μg/plate, respectively.

From the results, first, it can be concluded that the mutagenicity effect in the mixed test was from BP-1. Second, the positive dose was higher than those in the separate experiments, which indicated that the presence of BP decreased the mutagenicity of BP-1; therefore, BP and BP-1 showed antagonism in mutagenicity in the TA97 strain. There was some extent of dose–response (the reverse mutation colonies in 50% are almost three times more colonies than in 0.5%) effects, which were not detected in the separating tests. The result indicated the complexity in the mechanism of the mixed test. 

For the TA102 strain, a positive mutagenicity effect was detected in the separating tests by BP in which the doses were 0.05 and 0.5 μg/plate, respectively; however, there was no significant mutagenicity effect detected in the mixed tests. This result also indicated the antagonism in mutagenicity of BP and BP-1, similar to that in the TA97 strain. 

For the TA100 strain in a separating test, BP showed negative mutagenicity while BP-1 showed a positive mutagenicity effect at a dose of 0.5 μg/plate. The positive mutagenicity was observed in the mixed test at two concentrations with the doses of 3.5 and 7 μg/plate for BP and 14 and 28 μg/plate for BP-1. The dose of BP-1 in the mixture that produced a positive effect was higher than that in the separate experiments. This result shows antagonism in mutagenicity that was similar to the TA97 strain.

Generally speaking, BP and BP-1 showed antagonism in mutagenicity in the mixing test. In our previous studies about the ecotoxicity of the BP series of chemicals, a mixture of BP-3 and BP-4 showed antagonism to *Chlorella vulgaris* (algae), *Daphnia magna* (zooplankton), and *Brachydanio rerio* (fish) [4]. Furthermore, the mixtures of BP+BP-3 and BP+BP-4 showed antagonism to *D. magna* (unpublished data in our lab). The results about the mutagenicity of mixtures of BPs are similar to those from ecotoxicity tests. All the results show the complexity of the mixing effect.

All of the positive reverse mutation results occurred in the absence of the S9 liver extract. It may conclude that the mutagenicity comes from the BP chemicals themselves and their mixtures. The metabolic process may eliminate the mutagenicity of BP and BP-1.

At present, there are few studies on the mechanism of mixing toxicity of benzophenone series substances. The present research studied the effects of mixtures of BPs in mutagenicity. The toxicity of a chemical mixture is important to explain the behavior and overall impact of a single chemical in the environment. The mechanism of action of a single compound is also crucial for the analysis of mixed toxicity experimental results. Studies have shown that different combinations of chemicals may lead to different eco-toxic effects [3,4,11]. 

Although recent results showed that the concentration of BPs in the environment is low [24], most BPs are biologically persistent and cumulative and may cause damage to the aquatic environment and human health [3,4,11].

In the future, more research is expected to study the toxic effects of UV filters, as well as the effects of different combinations of mixes to explore the behavior of UV filters and effects on human health.

## 5. Conclusions

(1) The mutagenicity of BP and BP-1 was detected by the Ames assay. All the positive mutagenicity of BP and BP-1 was detected in the system without S9.
(2) In the separating tests with BP, positive mutations were detected in the TA102 strain at doses of 0.05 and 0.5 μg/plate.
(3) In the separating tests with BP-1, positive mutations were detected in the TA97 strain at doses of 0.05 and 0.5 μg/plate. In addition, positive mutations in the TA100 strain at a dose of 0.5 μg/plate were detected.
(4) In the mixed tests, the positive mutation results were found in strains TA97 and TA100. For the strain TA97, the positive mutation results were detected at 10% and 50%. For strain TA100, the positive mutation results were detected when the mixture was at 5% and 10%. All the mixture results showed antagonism in mutagenicity between BP and BP-1.

## Figures and Tables

**Table 1:** Reverse mutation colonies in the negative control and positive control groups.

Chemicals	Dose (μg/Plate)	TA97		TA98		TA100		TA102	
		−S9	+S9	−S9	+S9	−S9	+S9	−S9	+S9
Blank	0	144 ± 3	148 ± 7	33 ± 2	42 ± 2	157 ± 8	207 ± 5	227 ± 16	287 ± 6
DMSO	100	142 ± 3	144 ± 9	37 ± 3	38 ± 1	160 ± 10	195 ± 4	234 ± 15	287 ± 7
Dexon	50	1987 ± 203 *	-	1065 ± 137 *	-	-	-	586 ± 83 *	-
NaN_3_	1.5	-	-	-	-	912 ± 155 *	-	-	-
2-AF	10	-	1287 ± 168 *	-	1186 ± 154 *	-	896 ± 143 *	-	-
Dantron	50	-	-	-	-	-	-	-	625 ± 71 *

* MR ≥ 2 compared to control.

**Table 2:** Reverse mutation colonies of four *S.* *typhimurium* strains detected by BP.

Chemical	Dose (μg/Plate)	TA97		TA98		TA100		TA102	
		−S9	+S9	−S9	+S9	−S9	+S9	−S9	+S9
BP	0.05	123 ± 36	149 ± 4	36 ± 7	43 ± 13	156 ± 28	217 ± 25	1957 ± 57 *	343 ± 7
	0.5	119 ± 8	152 ± 8	43 ± 13	31 ± 4	190 ± 23	218 ± 8	1685 ± 173 *	365 ± 8
	5	114 ± 10	146 ± 13	27 ± 3	44 ± 4	140 ± 42	228 ± 21	255 ± 38	371 ± 8
	50	74 ± 13	147 ± 6	26 ± 4	46 ± 2	122 ± 15	233 ± 32	169 ± 16	377 ± 10
	500	3 ± 3	40 ± 14	23 ± 13	17 ± 2	0 ± 0	6 ± 6	0 ± 0	407 ± 9

* MR ≥ 2 compared to control.

**Table 3:** Reverse mutation colonies of four *S. typhimurium* strains detected by BP-1.

Chemical	Dose (μg/Plate)	TA97		TA98		TA100		TA102	
		−S9	+S9	−S9	+S9	−S9	+S9	−S9	+S9
BP-1	0.05	925 ± 13 *	151 ± 8	43 ± 13	51 ± 7	239 ± 17	174 ± 13	311 ± 12	350 ± 11
	0.5	697 ± 56 *	143 ± 10	46 ± 8	48 ± 2	458 ± 29 *	193 ± 12	369 ± 18	369 ± 10
	5	185 ± 10	146 ± 10	33 ± 3	52 ± 7	153 ± 2	191 ± 8	355 ± 20	443 ± 8
	50	83 ± 6	149 ± 11	22 ± 9	36 ± 1	188 ± 15	196 ± 4	407 ± 16	429 ± 8
	500	6 ± 1	88 ± 20	0 ± 1	23 ± 2	181 ± 10	122 ± 4	7 ± 4	390 ± 10

* MR ≥ 2 compared to control.

**Table 4:** Reverse mutation colonies of four *S. typhimurium* strains detected by the mixture of BP and BP-1.

Chemicals	Dose (Percentage %)	TA97		TA98		TA100		TA102	
		−S9	+S9	−S9	+S9	−S9	+S9	−S9	+S9
BP + BP-1 70 + 280 μg/mL	0.5	154 ± 8	159 ± 11	30 ± 8	39 ± 17	166 ± 9	178 ± 12	308 ± 31	284 ± 28
	5	213 ± 19	146 ± 10	29 ± 5	23 ± 19	478 ± 27 *	183 ± 6	298 ± 17	384 ± 19
	10	347 ± 21 *	163 ± 7	23 ± 3	30 ± 3	550 ± 30 *	190 ± 21	325 ± 18	398 ± 12
	50	450 ± 37 *	172 ± 15	48 ± 20	42 ± 4	210 ± 12	208 ± 33	242 ± 9	392 ± 9
	100	167 ± 11	153 ± 23	21 ± 9	27 ± 13	233 ± 17	191 ± 18	250 ± 28	316 ± 18

* MR ≥ 2 compared to control.

**Table 5:** Bacterial inhibition results of BP and BP-1 on four *S.* *typhimurium* strains.

Chemicals	Dose (μg/Plate)	*t*-Test Result			
		TA97	TA98	TA100	TA102
BP	50	−	−	−	−
	500	+	−	+	+
	1000	+	+	+	+
	2500	++	++	++	++
	5000	++	++	++	++
BP-1	50	−	−	−	−
	500	+	+	-	+
	1000	+	+	+	+
	2500	++	++	++	++
	5000	++	++	++	++

− not inhibit bacteria; + *t*-test (*p* < 0.05); ++ *t*-test (*p* < 0.01).

## Abbreviations

The following abbreviations are used in this manuscript:

BP	benzophenone
BP-1	benzophenone-1
PCPs	personal care products
